# Improving the outcomes from ruptured abdominal aortic aneurysm: interdisciplinary best practice guidelines

**DOI:** 10.1308/003588413X13511609956778

**Published:** 2013-03

**Authors:** RJ Hinchliffe, JT Powell

**Affiliations:** ^1^St George’s Healthcare NHS Trust, UK; ^2^Imperial College London, UK

**Keywords:** Ruptured abdominal aortic aneurysm, Emergency surgery, Vascular surgery

With increasing surgical specialisation and centralisation, some surgical emergencies can only be treated in specialist centres. In this context, guidelines for patient transfer from general to specialist hospitals can aid equity of patient access to emergency specialist surgical care. Guidelines have existed for some time on the early management and transfer of patients with head injury but for many surgical emergencies there have been no such guidelines.

Consequently, publication of joint Vascular Society of Great Britain and Ireland, College of Emergency Medicine and Royal College of Radiologists guidelines and audit standards on the diagnosis, early management and transfer of patients with ruptured abdominal aortic aneurysm (rAAA) from general hospitals is welcome.^1^ If widely adopted, the guidelines will optimise patient care and have the potential to save lives. Unfortunately, guidelines have not existed for vascular emergencies such as rAAA. Data from Europe suggest that streamlined regional pathways do improve patient outcomes from rAAA.^2^ Interestingly, these guidelines were stimulated through research, when it became apparent during the IMPROVE (Immediate Management of the Patient with Ruptured aneurysm: Open Versus Endovascular repair) trial^3^ that practices to transfer patients with rAAA to a specialist centre varied widely across England. This led to a three-round Delphi consensus approach to examine the areas of consensus and disagreement concerning the transfer of patients with rAAA.^4^

RAAA is a lethal condition in which many patients die before they reach hospital. An appropriately trained vascular team including experienced vascular surgeons has the potential to improve outcomes of those reaching a vascular unit alive. This is dependent, in large part, on rapid diagnosis, appropriate early management and swift transfer. The diagnosis can be difficult, especially in the elderly with multiple co-morbidities.

The development of a vascular surgical specialty, with its own specialist training, and the centralisation of vascular services in the UK will result in an increasing number of hospitals being unable to provide onsite operative intervention for rAAA. Consequently, increasing numbers of patients will have to be assessed locally by other specialists (many of whom will not have managed patients with rAAA), who will have to institute immediate care and consider which patients they should transfer to a specialist vascular centre. However, there are changes that might contribute to patient management. Emergency medicine trainees are now trained to perform abdominal ultrasonography to identify a number of key pathologies, including AAA.

In the UK and elsewhere, patients have had variable quality of care, largely owing to inequality of access to specialist vascular surgical assessment and treatment when they present but also owing to an absence of standardisation of preoperative assessment and early management. General hospitals that operate on just a handful of patients with rAAA each year tend to have a higher mortality rate than that of specialist vascular centres.^5,6^ The elderly and women have been notably discriminated against.^7,8^

The Delphi consensus study on the transfer of patients from district general hospitals to vascular centres analysed ten areas of process of care from the diagnosis of rAAA through to the necessary conditions in the receiving hospital.^4^ The study demonstrated broad agreement about the type of patient who should be eligible for transfer but disagreements about patient management before and during transfer. Subsequently, in collaboration with the College of Emergency Medicine and others, the guidance document was developed. This document contains six key steps, which are summarised below. They are pragmatic to ensure that patients have rapid diagnosis, appropriate immediate management to prevent deterioration, and rapid and equitable access to expert vascular care.

A clinical diagnosis of rAAA by an experienced emergency department doctor is sufficient to transfer patients to a vascular centre. The use of ultrasonography by trained personnel is useful if the presence of an AAA is suspected (but ultrasonography cannot make the diagnosis of rupture). Computed tomography (CT) is helpful in the diagnosis of rAAA but should not delay patient transfer. It is better to perform CT in the vascular centres rather than delay transfer.

An assessment of premorbid conditions (including advanced directive) should be performed in all patients. It is inappropriate to transfer patients who have suffered a cardiac arrest in this illness. The other guidance is pragmatic. In very frail patients or where there are other concerns, consultant involvement in the discussion is helpful.

Patients at the general hospital do not require assessment by a general surgeon. An experienced emergency department doctor is sufficient to make the diagnosis and refer patients.

All patients should have continuous vital sign monitoring during assessment and transfer. Investigations should not delay the transfer and none are essential. Intravenous access is the only necessary invasive procedure required. Permissive hypotension should be instituted with small boluses of fluids given only if necessary to maintain a systolic blood pressure of >70mmHg. Aggressive fluid resuscitation should be avoided.

The transfer of the patient should be as swift as possible. The next available ambulance (‘time critical transfer’) is appropriate. A paramedic crew is preferred but not essential. Any imaging should be transferred with the patient or electronically (not allowed to delay). No blood should be transferred with the patient (as it cannot be used at the receiving vascular unit).

Patients should be transferred to an area that offers critical care facilities. This will depend on the local arrangements and the ambulance should be informed of the destination prior to transfer. After agreement to accept the patient, the vascular consultant/senior trainee should put in place all necessary arrangements to manage the patient expeditiously on arrival. It is expected that formal protocols of care will exist in the vascular unit to facilitate management of patients with rAAA (although this is beyond the scope of the guidance).

An audit standard has been introduced with the new guidelines. All appropriate patients with rAAA should be transferred to specialist vascular care within 30 minutes of the diagnosis of rAAA being made.

RAAA is a good example of a surgical emergency where patients stand to benefit from the introduction of guidelines and audit standards.
